# Roles of long non-coding RNA *HULC* in human digestive system cancers

**DOI:** 10.3389/fonc.2025.1642425

**Published:** 2025-08-20

**Authors:** Liqing Huang

**Affiliations:** Three Gorges University Hospital of Traditional Chinese Medicine & Yichang Hospital of Traditional Chinese Medicine, Yichang, China

**Keywords:** lncRNA, HULC, digestive system cancers, cancer, ceRNA

## Abstract

Digestive system cancers, including hepatocellular carcinoma (HCC), gastric cancer (GC), pancreatic cancer (PC), and colorectal cancer (CRC), pose a significant global health burden with high morbidity and mortality rates. Their tumorigenesis and progression are driven by complex interactions between genetic alterations and environmental factors. In recent years, long non-coding RNAs (lncRNAs) have emerged as critical regulators in cancer initiation, metastasis, and drug resistance through epigenetic modulation, transcriptional regulation, and post-transcriptional modifications. Among them, HULC, a well-characterized oncogenic lncRNA, was initially identified in HCC due to its remarkable upregulation. Subsequent studies have revealed that HULC is aberrantly overexpressed in multiple gastrointestinal malignancies, including GC, PC, and CRC, and its expression levels strongly correlate with advanced clinical stage, metastatic potential, and poor patient prognosis. Mechanistically, HULC exerts its oncogenic effects by interacting with genes, RNA, and proteins to promoting the Warburg effect, and inducing epithelial-mesenchymal transition (EMT), thereby facilitating tumor progression. This review comprehensively summarizes recent advances in understanding the biological roles, molecular mechanisms, and clinical implications of HULC in digestive system cancers. Furthermore, we discuss its potential as a novel diagnostic biomarker and therapeutic target, providing insights into precision medicine strategies for gastrointestinal malignancies.

## 1 Introduction

Digestive system tumors, mainly including hepatocellular carcinoma (HCC), pancreatic cancer (PC), gastric cancer (GC), and colorectal cancer (CRC), represent a major cause of cancer-related morbidity and mortality worldwide. According to recent statistics, more than 5 million new cases are diagnosed annually, resulting in approximately 4 million deaths globally. This imposes a substantial economic burden on patients, families, and healthcare systems (1, 2). Despite the availability of diverse treatment modalities, including surgery, chemotherapy, radiotherapy, immunotherapy and targeted therapies, therapeutic efficacy remains limited, particularly for patients with advanced or metastatic disease (3, 4). Consequently, the identification of novel molecular targets is urgently needed to improve diagnostic precision and develop more effective treatment strategies for digestive system cancers.

Long non-coding RNAs (*lncRNAs*), a class of non-protein-coding transcripts longer than 200 nucleotides, are transcribed by RNA polymerase II and have emerged as key regulators in cancer biology (5). Increasing evidence indicates that *lncRNAs* play critical roles in tumor initiation, proliferation, invasion, and prognosis through various mechanisms, including epigenetic modification, chromatin remodeling, and post-transcriptional regulation (6–9). Among these mechanisms, the competitive endogenous RNA (*ceRNA*) network is particularly noteworthy. In this context, *lncRNAs* act as molecular sponges by binding to microRNAs(*miRNAs*), thereby modulating the expression of downstream target genes. This *ceRNA*-mediated regulatory axis is now recognized as a significant contributor to tumor progression (5, 10, 11).

*HULC* is located on chromosome 6p24.3 (12). Following transcription and post-transcriptional processing, a mature lncRNA is produced (12). Increasing evidence suggests that *HULC* is aberrantly expressed in various gastrointestinal cancers, including hepatocellular carcinoma, gastric cancer, and colorectal cancer (13–15). Additionnally, *HULC* has also been shown to promote the progression of other systemic cancers, including breast cancer, lung cancer, ovarian cancer, and osteosarcoma (16–19).For instance, *HULC* promotes the metastasis and cisplatin resistance of triple-negative breast cancer cells by targeting the trans-IGF1R-PI3K-AKT axis35981570. Additionally, *HULC* enhances the proliferation of lung squamous cancer cells by regulating the PTPRO/NF-κB signaling pathway (17). However, in comparison to non-gastrointestinal cancers, *HULC* shows a stronger correlation with digestive system cancers (20, 21). Furthermore, *HULC* has been studied most extensively within the context of digestive system cancers.Therefore, we have decided to focus on digestive system tumors to thoroughly investigate the biological functions and clinical significance of *HULC* in this area.

The expression of *HULC* expression is regulated through multiple molecular mechanisms. For instance, *HULC* can interact with IGF2 mRNA-binding protein 1 (IGF2BP1), leading to reduced *HULC* stability and promoting transcript degradation (22). Additionally, *miR-203* has been shown to participate in the post-transcriptional regulation of *HULC* (23). In this review, we summarize the expression patterns, biological functions, and molecular mechanisms of *HULC* in digestive system cancers, and discuss its potential as a novel diagnostic biomarker and therapeutic target. The current body of evidence highlights the pivotal role of *HULC* in the initiation, progression, and metastasis of digestive system tumors, underscoring its promise as a target for future precision medicine strategies.

## 2 Characterization of *HULC*

*HULC* is located on chromosome 6, spanning positions 8,435,568 to 9,294,133 on the human genome reference sequence GRCh38.p14. According to Ensembl websites, there are a total of 217 transcripts(splice variants) for *HULC*. Among them, the transcript *lncRNA HULC-202*(Ensembl transcript ID: ENST00000503668.3; NCBI transcript ID: NR_004855.3) is the most well-characterized transcript, with a length of 434 base pairs. Additionally, *lncRNA HULC*-202 has been studied across various types of cancers, including liver, gastric, and colorectal cancer (24–26). Accumulating evidence shows that *HULC* is aberrantly expressed in various gastrointestinal malignancies, including liver, gastric, and colorectal cancers (13–15). Although the physiological functions and molecular mechanisms of *HULC* are not yet fully understood, current findings can be summarized into the following major functional roles:

1. HULC and gene activation and inhibition: HULC regulates the histone modification pattern in the promoter region of the *YAP* gene by increasing the enrichment of H3K4me3 and reducing the enrichment of H3K27me3. Under hyperglycemic conditions, HULC promotes the transcriptional activation of the *YAP* gene, which is linked to the proliferation of pancreatic cancer cells and enhanced drug resistance (27). Furthermore, HULC may also influence the expression of neighboring genes. In radiation-induced liver cancer, HULC was found to downregulate the expression of the nearby gene *CDKN1* through complementary base pairing, thereby affecting tumor progression. Additionally, Li Dan et al. reported that downregulation of HULC expression in hepatoma cell lines such as Hep3B and HepG2 led to reduced expression of the adjacent gene *SLC35B3* (28).
2. The interaction between HULC and RNA(Acting as a ceRNA): HULC functions as a ceRNA by sharing miRNA binding sites with target transcripts. Through this mechanism, *HULC* competitively binds miRNAs, thereby mitigating their inhibitory effects on downstream target gene expression. This is currently the most extensively studied mechanism. For example, in liver cancer, *HULC* directly binds to *miR-372*, leading to *miR-372* decreased expression and activity. In turn, *miR-372* normally reduces the phosphorylation of cAMP response element-binding protein (CREB), thus diminishing CREB’s binding to the *HULC* promoter and lowering *HULC* transcription. Consequently, a positive feedback loop is established, further enhancing *HULC* expression (29). Moreover, *HULC* promotes autophagy through the *miR-675*/PKM2 axis, leading to upregulation of Cyclin D1 and accelerated proliferation of liver cancer stem cells (30). Similarly, via the *miR-9*/PPARA signaling pathway, *HULC* activates ACSL1 and induces abnormal lipid metabolism in liver cancer cells, contributing to disease progression (31). In gastric cancer, *HULC* promotes cell proliferation, migration, invasion and resistance to apoptosis through the *miR-9-5p*/MYH9 axis (25).
3. The interaction between *HULC* and proteins: Lactate dehydrogenase A (LDHA) and pyruvate kinase M2 (PKM2) are two key enzymes involved in glycolytic reprogramming, a hallmark of cancer that promotes rapid cell growth and survival. Studies have demonstrated that *HULC* can directly bind to and increase the phosphorylation of both LDHA and PKM2, thereby enhancing glycolysis in HCC cell lines and facilitating tumor progression (32). Furthermore, *lncRNA MEG3* promotes the binding of the p53 protein to *HULC*, influencing the interaction between the telomere length maintenance complex and telomeric DNA, which leads to reduced telomere stability (33).
4. Overall, *HULC* interacts with genes, RNA, and proteins to promote tumor cell metabolism reprogramming(Warburg effect), and an anti-apoptotic phenotype. Ultimately, it contributes to epithelial-mesenchymal transition (EMT), invasion and metastasis, and immune escape in cancers.

## 3 *HULC* in digestive tumors

**Figure 1:**
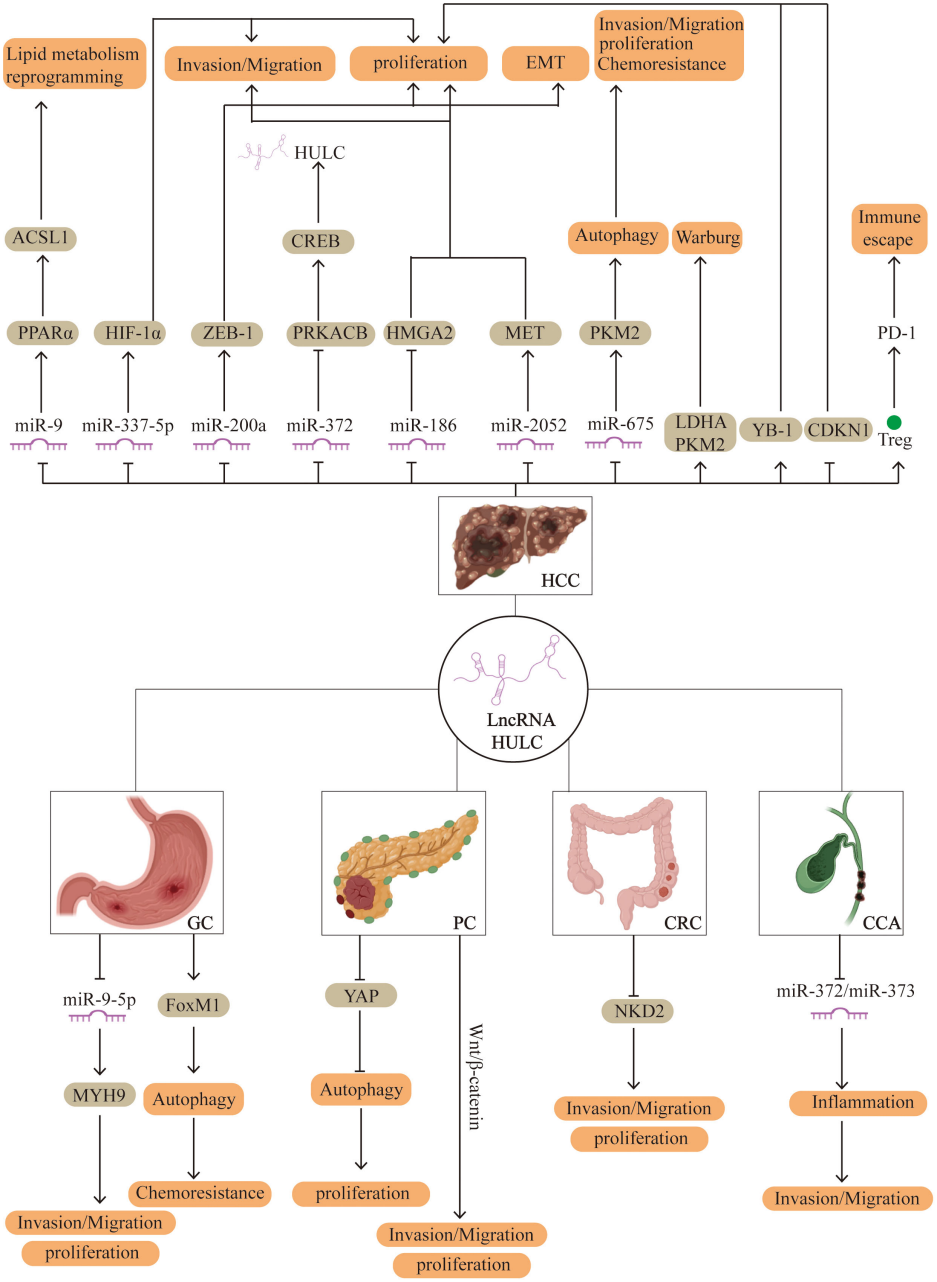
Potential regulatory mechanisms of *HULC* in human digestive system cancer. HCC, hepatocellular carcinoma; PC, pancreatic cancer; GC, gastric cancer; CRC, colorectal cancer; CCA, Cholangiocarcinoma.

Recent studies have highlighted the pivotal role of lncRNAs in the pathogenesis of digestive system cancers. Aberrant expression of lncRNAs has been implicated in key oncogenic processes such as cell proliferation, invasion, metastasis, epithelial-mesenchymal transition, and resistance to apoptosis. As research has progressed, *HULC* has been found to be abnormally expressed in a range of digestive system malignancies, where it contributes to the regulation of tumor cell behavior (**Figure 1**).

### 3.1 *HULC* in hepatocellular carcinoma

#### 3.1.1 Aberrant up‐regulation of *HULC*

In 2007, Panzitt et al. first identified the abnormal expression of *HULC* in HCC using genome-wide microarray analysis (12). This study included 46 HCC tissues, 4 liver focal nodular hyperplasia samples, 7 liver cirrhosis samples, and 2 normal liver samples. The results demonstrated a gradual upregulation of *HULC* expression across liver cirrhosis, focal nodular hyperplasia, and ultimately HCC. These findings have been consistently validated in subsequent studies (22, 34, 35). Furthermore, radioactive *in situ* hybridization confirmed the elevated expression of *HULC* in liver cancer tissues (12). In addition, quantitative real-time polymerase chain reaction (qRT-PCR) has shown significantly increased *HULC* levels in HCC cell lines (29, 36). A clinical study conducted in Egypt further supported these findings, revealing that *HULC* expression in the blood of HCC patients was significantly higher than in patients with chronic hepatitis C virus (HCV) infection (35). These collective data suggest that *HULC* may serve as a novel biomarker for the early diagnosis of HCC. A meta-analysis involving 6,426 HCC patients indicated that *HULC* exhibits superior sensitivity and specificity for HCC diagnosis compared with traditional biomarkers or other non-coding RNAs (37). Moreover, elevated serum *HULC* levels are associated with poor prognosis and have clinical utility in predicting metastasis and outcomes after radical resection of HCC (38, 39).

Notably, Yao et al. recently developed a probe-based rolling circle amplification-induced fluorescence biosensor for detecting *lncRNA HULC*. This biosensor demonstrated high selectivity and sensitivity in both HCC cell lines and whole blood samples from HCC patients (40). These advancements highlight a promising new approach for the clinical application of *HULC* in the diagnosis, prognosis, and potentially treatment monitoring of HCC.

#### 3.1.2 Mechanism of *HULC* in hepatocellular carcinoma

Studies have demonstrated that *HULC* plays a critical role in promoting the progression of liver cancer by interacting with genes, RNA, and proteins. This section summarizes the key regulatory mechanisms in HCC driven by *HULC*.

##### 3.1.2.1 *HULC/miRNA-9*/PPARA

Aberrant lipid metabolism has been recognized as a key contributor to malignant tumor progression. Long-chain acyl-CoA synthetase 1 (ACSL1), a critical enzyme involved in cholesterol biosynthesis and fatty acid oxidation, plays a pivotal role in these metabolic alterations. Clinical studies in patients with HCC have shown that *HULC* expression is positively correlated with ACSL1 expression, as well as with serum triglyceride and cholesterol levels. Supporting these findings, *in vitro* experiments demonstrated that *HULC* activates ACSL1 expression in HepG2 hepatoma cells via the *miRNA-9*/PPARA signaling pathway, thereby inducing lipid metabolic reprogramming that facilitates HCC progression. Moreover, elevated intracellular cholesterol levels may further amplify *HULC* expression through activation of retinoid receptors in hepatoma cells, establishing a positive feedback loop that exacerbates tumor development (22).

##### 3.1.2.2 *HULC/miR-377-5p*/HIF-1α

In the HCC cell lines, including HB611, H22 and HepG2, qRT-PCR showed significant increases in both *HULC* expression, while *miR-377-5p* expression was decreased. Inhibition of *HULC* expression in HepG2 cells suppressed both cell proliferation and invasion. Conversely, *miR-377-5p* inhibition promoted hepatoma cell proliferation and invasion. Further experiments suggested that *HULC* may promote HCC progression by directly targeting the *miR-377-5p*/HIF-1α pathway (36).

##### 3.1.2.3 *HULC* in HBV-associated HCC

chronic hepatitis B virus (HBV) infection is a major cause of cirrhosis and HCC. Hepatitis B virus X protein (HBx) is the key pathogenic factor in HBV infection. Luciferase reporter gene and chromatin immunoprecipitation assays have demonstrated that HBx activates the *HULC* promoter via cAMP response element-binding protein (CREB), thereby promoting *HULC* expression (41). Further studies revealed that HBx enhances hepatoma cell proliferation by upregulating the *HULC*/p18 pathway (41). In HBV-related HCC, metformin has been shown to inhibit tumor cell proliferation by negatively regulating the *HULC*/p18/miR-200a/ZEB1 signaling pathway. This regulation improves survival rates and reduces recurrence in HCC patients (42). Moreover, upregulation of *HULC* promotes the HBx/STAT3/*miR-539*/APOBEC3B pathway, which further supports HBV replication and accelerates hepatoma cell growth (43).

##### 3.1.2.4 HULC/miR-372

Luciferase assays indicated that *miR-372* reduced the promoter activity of *HULC* in Hep3B hepatoma cells. Overexpression of *miR-372* inhibited the translation of its target gene PRKACB, leading to a decrease in CREB phosphorylation. This reduction impaired the binding of CREB to the proximal *HULC* promoter, thereby lowering *HULC* expression. Conversely, *HULC* inhibits *miR-372* activity, leading to upregulation of *HULC* expression in liver cancer (29).

##### 3.1.2.5 HULC/miR-186

High-mobility group protein A2 (HMGA2), a transcription factor, is involved in various cancers, including liver, colorectal, and gastric cancers. In HCC tissues, qRT-PCR analysis revealed a positive correlation between *HULC* and HMGA2 expression. *In vitro* studies demonstrated that *HULC* upregulates HMGA2 expression in HCC cells. Further investigation showed that *HULC* promotes the expression of HMGA2 by inhibiting *miR-186*, thereby facilitating liver cancer progression (44).

##### 3.1.2.6 HULC/miR-2052

Bioinformatics analyses suggest that *HULC* acts as a sponge for *miR-2052*. In 42 pairs of HCC and matched non-cancer tissues, the expression of *miR-2052* was negatively correlated with *HULC* levels. Both *in vitro* and *in vivo* experiments confirmed that *HULC* promotes HCC cell proliferation, migration, invasion, and progression through the *miR-2052*/MET axis (45).

##### 3.1.2.7 *HULC* and autophagy

Autophagy plays a critical role in the initiation and progression of HCC, and *HULC* has emerged as a key modulator of this process. In liver cancer stem cells, *HULC* was shown to induce autophagy via the *miR-675*/PKM2 pathway, leading to upregulation of Cyclin D1 and promoting the proliferation of liver cancer stem cells (30). Additionally, *HULC* activates autophagy by enhancing the expression of LC3-I and LC3-II (canonical markers of autophagy) through the deacetylase Sirt1 (24). Further studies by Liu et al. demonstrated that *HULC* also promotes phosphorylation of p65 and IκBκB, thereby increasing LC3-II levels in an NF-κB-dependent manner (46). Mechanistically, *HULC* inhibits PTEN via autophagy-mediated degradation through the ubiquitin-proteasome system, which in turn activates the PI3K/AKT/mTOR pathway to promote hepatocarcinogenesis (24). Moreover, autophagy contributes to chemoresistance in HCC. Anti-tumor agents such as oxaliplatin, 5-fluorouracil, and pirarubicin have been observed to increase both *HULC* expression and autophagic activity in human HCC tissues. *In vivo* data indicate that *HULC* overexpression reduces HCC cell sensitivity to oxaliplatin, whereas silencing *HULC* enhances drug sensitivity. Further *in vitro* experiments revealed that the USP22/Sirt1/autophagy axis underlies this effect, suggesting that targeting this pathway could provide a novel therapeutic approach to overcoming chemoresistance in HCC (47, 48).

##### 3.1.2.8 *HULC* and Warburg effect

Warburg effect is a hallmark of cancer metabolism wherein cancer cells preferentially undergo glycolysis over oxidative phosphorylation, even under normoxic conditions. This metabolic shift supports rapid tumor growth and survival. *HULC* has been found to bind directly to and increase the phosphorylation of key glycolytic enzymes LDHA and PKM2, thereby promoting glycolysis and enhancing tumor progression (32).

##### 3.1.2.9 *HULC* and EMT

EMT is essential for tumor invasion and metastasis. Clinical data reveal that *HULC* expression is positively correlated with EMT phenotypes in HCC. *In vitro* studies further demonstrated that *HULC* upregulates EMT markers such as N-cadherin and vimentin. Mechanistically, *HULC* induces EMT via the *miR-200a-3p*/ZEB1 signaling pathway, thereby facilitating tumor progression and metastasis (49).

##### 3.1.2.10 *HULC*/YB-1

*HULC* interacts with Y-box-binding protein 1 (YB-1), a multifunctional protein involved in mRNA splicing, translation regulation and DNA repair. Mass spectrometry, localization, and co-immunoprecipitation studies revealed that *HULC* binds YB-1 and promotes its phosphorylation via extracellular signal-regulated kinase signaling. This phosphorylation leads to the release of YB-1 from silenced oncogenic mRNAs, including cyclin D1, cyclin E1, and matrix metalloproteinase 3, thereby enhancing their translation and accelerating tumor growth (50).

##### 3.1.2.11 *HULC*/CDKN1

In radiation-induced liver carcinogenesis, *HULC* also plays a significant role. Downregulation of *HULC* impairs hepatocyte proliferation following radiation exposure. CDKN1, a gene located adjacent to *HULC*, has been implicated in radiation-induced cell cycle regulation. *In vitro* studies indicate that *HULC* suppresses CDKN1 expression via complementary base pairing, thereby promoting cell cycle progression and contributing to radiation-related liver cancer (51).

##### 3.1.2.12 *HULC*/Treg/PD-1

Immune escape represents a major challenge in malignant tumor therapy. In a liver cancer xenograft model, overexpression of *HULC* increased regulatory T cell (Treg) proliferation and upregulated PD-1 expression in the tumor microenvironment. Additional studies revealed that the *HULC*–Treg–PD-1 axis suppresses IL-10 and TGF-β1 expression, facilitating immunosuppression and promoting immune escape. Targeting this axis may represent a promising strategy to overcome immune resistance in HCC (52).

Collectively, these findings underscore the multifaceted role of *HULC* in hepatocellular carcinoma. *HULC* expression is significantly elevated in HCC, especially in cases with focal nodular hyperplasia and liver cirrhosis, highlighting its potential as a diagnostic biomarker. It promotes HCC initiation, progression, metastasis, and therapy resistance by modulating key mechanisms such as interactions with multiple miRNAs/transcription factors, autophagy, the Warburg effect, EMT, and immune escape. Inhibition of *HULC* and its downstream pathways may offer new therapeutic opportunities for HCC treatment and prognosis improvement.

### 3.2 *HULC* in gastric cancer

Multiple clinical studies have demonstrated that *HULC* is significantly overexpressed in both plasma and tissue samples from patients with GC compared to healthy controls. Survival analyses have shown that elevated *HULC* expression is significantly associated with poorer overall survival (53, 54). In addition, circulating *HULC* levels correlate with tumor size, lymph node involvement, distant metastasis, and Helicobacter pylori infection in GC patients (13, 55). Notably, the combination of *HULC* expression and H. pylori status improves the predictive accuracy for GC risk (56). These findings suggest that *HULC* is a promising biomarker for GC diagnosis and prognosis.

*In vivo*, silencing *HULC* significantly inhibited tumor growth in a mouse xenograft model of gastric cancer (25). *In vitro*, overexpression of *HULC* promotes the proliferation and invasion of human gastric cancer cells, while inhibiting cell apoptosis. Conversely, knockdown of *HULC* reverses these effects (57). Mechanistically, dual-luciferase reporter assays and RNA pull-down experiments revealed that *HULC* directly targets *miR-9-5p* to exert its biological functions. Further studies have demonstrated that *HULC* enhances proliferation, EMT, migration, and invasion of gastric cancer cells through the *miR-9-5p*/MYH9 axis (25, 57). Additionally, *HULC* promotes gastric cancer growth and metastasis by epigenetically suppressing the expression of p53. Specifically, RIP- and ChIP-qPCR assays have shown that *HULC* recruits EZH2 to the p53 promoter region, mediating its transcriptional repression in gastric cancer cells (58). Moreover, *HULC* plays a critical role in regulating chemoresistance. Inhibition of *HULC* enhances cisplatin-induced apoptosis in gastric cancer cells (47), and the *HULC*/FoxM1 signaling pathway has been shown to promote cisplatin resistance by inducing autophagy (52).

Taken together, these findings highlight *HULC* as an important regulator of tumor growth, metastasis, and drug resistance in gastric cancer. *HULC* represents a promising biomarker for early detection and prognosis and may serve as a novel therapeutic target in the prevention and treatment of GC.

### 3.3 *HULC* in pancreatic cancer

Pancreatic cancer ranks as the fourth leading cause of cancer-related deaths in the United States and the sixth in China (14, 27). Bioinformatics analyses have identified *HULC* as one of the most significantly dysregulated lncRNAs in pancreatic ductal adenocarcinoma cells (27). A clinical study involving tumor and matched normal tissues from 304 PC patients revealed that *HULC* is highly expressed in both the tissues and serum of PC patients. Notably, elevated *HULC* levels were significantly associated with larger tumor size, lymph node metastasis, and vascular invasion.

*In vivo*, using PC xenografts in nude mice demonstrated that *HULC* knockdown significantly suppressed tumor growth compared to controls (59). Complementary *in vitro* studies further showed that silencing *HULC* reduced pancreatic ductal adenocarcinoma cell proliferation, viability, invasion, and migration. *HULC* knockdown also led to decreased expression of EMT markers(N-cadherin, vimentin, and Snail), while increasing the expression of E-cadherin (59). Mechanistically, *HULC* appears to promote pancreatic ductal adenocarcinoma progression via multiple pathways. It inhibits YAP activation, thereby promoting autophagy and enhancing tumor cell proliferation (27). Additionally, *HULC* may facilitate pancreatic ductal adenocarcinoma cell proliferation and invasion through the Wnt/β-catenin signaling pathway (14). *HULC* also plays a role in intercellular communication through extracellular vesicles, where it promotes invasion and migration by inducing EMT in recipient cells. This effect can be counteracted by miR-622, which targets *HULC* to suppress EMT-related signaling, providing new insights into pancreatic ductal adenocarcinoma pathogenesis and potential therapeutic targets (60).

### 3.4 *HULC* in colorectal carcinoma

Colorectal cancer is one of the main causes of cancer deaths worldwide. By bioinformatics analysis, lncRNA *HULC* is regarded as a potential biomarker for colorectal cancer (2, 61). Additionally, previous research has demonstrated that serum *HULC* levels are significantly elevated in patients with CRC compared to healthy individuals. This elevation suggests that *HULC* may serve as a promising biomarker for the diagnosis of CRC (15). *HULC* expression was significantly upregulated in human primary colorectal cancer and colorectal cancer cell lines. Kaplan-Meier survival analysis showed that the upregulation of *HULC* was significantly associated with poor prognosis in patients with colorectal cancer. Knockdown of *HULC* significantly inhibited the proliferation, migration and invasion of CRC cells and promoted cell apoptosis *in vitro*. In BALB/c nude mice tumorigenic experiments show that knockdown of *HULC* can inhibit the proliferation of CRC cells. Mechanically speaking, RNA immunoprecipitation (RIP) and RNA pull-down experiments indicated that in colorectal cancer cells, *HULC* promoted CRC by directly binding to EZH2 to inhibit the expression of NKD2 (62).

### 3.5 Others

Cholangiocarcinoma (CCA) is a highly fatal malignancy with a poor overall survival rate. Bioinformatics analysis of 36 CCA tumor tissues and 9 normal control tissues from WMU cohort revealed that *HULC* was significantly upregulated and strongly associated with shorter overall survival in CCA patients (63). Functional assays, including cell migration, and invasion experiments, demonstrated that *HULC* overexpression enhances the migratory and invasive capacities of CCA cells. Further mechanistic studies showed that *HULC* acts by targeting *miR-372/miR-373*, leading to upregulation of inflammation-related genes such as *IL-6* and *CXCR4*. This, in turn, induces aberrant inflammatory responses and promotes cancer cell migration and invasion (64).

*HULC* is also implicated in the pathogenesis of oral squamous cell carcinoma (OSCC), one of the most prevalent malignancies in the oral and maxillofacial region worldwide (65). In a murine xenograft model, depletion of *HULC* reduced tumor growth and suppressed EMT. *In vitro*, *HULC* knockdown inhibited proliferation, migration, and invasion of OSCC cells, while increasing apoptosis (66). These findings suggest that *HULC* plays a key role in OSCC tumorigenesis and progression.

In nasopharyngeal carcinoma (NPC), clinical studies have shown that *HULC* is highly expressed in tumor tissues and is associated with poor patient prognosis. *HULC* overexpression promotes the growth of NPC cells, whereas its downregulation activates the tumor suppressor p53, increases p21 expression, and induces cell cycle arrest and apoptosis (67). These data support a role for *HULC* as a carcinogenic lncRNA and highlight its potential as a therapeutic target in NPC.

Although *HULC* has been strongly implicated in the development and progression of several digestive system cancers, its role in esophageal cancer remains unclear. From 2014 to 2018, comparative analyses of tumor tissues from 95 patients with esophageal cancer and 121 healthy control samples showed no significant association between *HULC* expression and cancer prognosis across any clinical subgroup (68, 69). This suggests that the contribution of *HULC* to esophageal cancer may be limited or context-dependent, warranting further investigation.

## 4 Conclusion

*HULC* has emerged as a critical molecule in the progression of various digestive system malignancies, including hepatocellular carcinoma, pancreatic cancer, gastric cancer, and colorectal cancer liver metastasis. Multiple studies have shown that *HULC* is significantly upregulated in various digestive system cancers. Its abnormal upregulation is thought to result from a complex interplay between environmental factors (such as HBV infection) and intrinsic cellular dysregulation involving transcription factors and miRNAs.Importantly, the overexpression of *HULC* is associated with poor prognosis, highlighting its potential as a prognostic biomarker and therapeutic target. Mechanistically, *HULC* induces malignant phenotypes such as Warburg effect and EMT in tumor cells through interactions with genes, RNA, and proteins. These mechanisms do not exist in isolation but have extensive interactions. Therefore, as a promising therapeutic target, *HULC* has the following advantages: 1) High tissue specificity: *HULC* is significantly upregulated in digestive system tumors and is expressed at a lower level in most normal tissues, minimizing potential off-target effects. 2) Multimechanism carcinogenicity: It mediates the Warburg effect, EMT, and resistance to cell apoptosis through multiple mechanisms, making it a core node in combined therapy. 3) Non-coding features: As an LncRNA, *HULC* is less prone to mutation than protein-coding genes, reducing the risk of developing treatment resistance through genetic variations. 4) Detectability in liquid biopsy: *HULC* is elevated and stable in the blood of digestive system cancers, facilitating non-invasive monitoring and early intervention. Currently, the related mechanism studies of *HULC* in liver cancer are the most in-depth. In pancreatic cancer, gastric cancer, and colorectal cancer and other digestive system cancers, the research on *HULC* is relatively less. This suggests two possible interpretations: either *HULC* may warrant further investigation in other digestive system cancers, or it may possess higher specificity and clinical utility in liver cancer. Notably, clinical evidence indicates that *HULC* is associated with nearly all digestive system cancers, with the exception of esophageal cancer. Therefore, further research on *HULC* is crucial for comprehensively elucidating its biological function and clinical application.

## 5 Limitation

### 5.1 Heterogeneity among patients and tissues

Digestive system cancers exhibit tumor heterogeneity, both across different cancer types and among individual patients. This heterogeneity complicates the interpretation of findings and may affect the generalizability of results. Specifically, the expression levels and functional roles of *HULC* can differ significantly among patients. To elucidate the broader patterns and clinical relevance of *HULC*, larger, well-powered studies are necessary. In future research, machine learning algorithms may be employed to construct predictive models capable of identifying *HULC* expression signatures from diverse patient-derived samples.

### 5.2 Complexity of mechanisms

*HULC* exerts its effects through multiple molecular interactions, involving genes, RNA, and proteins. These interactions include diverse epigenetic mechanisms such as DNA methylation, histone modifications, and regulation by non-coding RNAs. The diversity of these epigenetic processes present a substantial challenge for studying the potential mechanisms of *HULC* in tumorigenesis. To address this, future studies should incorporate high-throughput sequencing, single-cell analysis, multi-omics integration, and large-scale sample validation, along with investigations into newly discovered epigenetic modifications.

### 5.3 Methodological challenges in studying *HULC*

Accurate detection and quantification of *HULC* require highly sensitive and specific techniques, which depend on sophisticated laboratory infrastructure and skilled personnel to ensure data quality and reproducibility. Furthermore, high-throughput technologies generate vast amounts of data, the analysis of which demands advanced bioinformatics tools and expertise. Extracting biologically meaningful insights from these datasets remains a critical challenge. Future investigations should leverage cutting-edge sequencing platforms and innovative functional assays to elucidate the oncogenic role and clinical significance of *HULC* in digestive system cancers.

## References

[1] Assarzadegan N Montgomery E. What is new in the 2019 world health organization (Who) classification of tumors of the digestive system: review of selected updates on neuroendocrine neoplasms, appendiceal tumors, and molecular testing. Arch Pathol Lab Med. (2021) 145:664–77. doi: 10.5858/arpa.2019-0665-RA, PMID: 32233993 PMC9281538

[2] Sung H Ferlay J Siegel RL Laversanne M Soerjomataram I Jemal A. Global cancer statistics 2020: globocan estimates of incidence and mortality worldwide for 36 cancers in 185 countries. CA Cancer J Clin. (2021) 71:209–49. doi: 10.3322/caac.21660, PMID: 33538338

[3] Lee YT Tan YJ Oon CE. Molecular targeted therapy: treating cancer with specificity. Eur J Pharmacol. (2018) 834:188–96. doi: 10.1016/j.ejphar.2018.07.034, PMID: 30031797

[4] Hojman P Gehl J Christensen JF Pedersen BK. Molecular mechanisms linking exercise to cancer prevention and treatment. Cell Metab. (2018) 27:10–21. doi: 10.1016/j.cmet.2017.09.015, PMID: 29056514

[5] Bhan A Soleimani M Mandal SS. Long noncoding rna and cancer: A new paradigm. Cancer Res. (2017) 77:3965–81. doi: 10.1158/0008-5472.CAN-16-2634, PMID: 28701486 PMC8330958

[6] Hu Y Wang J Qian J Kong X Tang J Wang Y. Long noncoding rna gaplinc regulates cd44-dependent cell invasiveness and associates with poor prognosis of gastric cancer. Cancer Res. (2014) 74:6890–902. doi: 10.1158/0008-5472.CAN-14-0686, PMID: 25277524

[7] Kogo R Shimamura T Mimori K Kawahara K Imoto S Sudo T. Long noncoding rna hotair regulates polycomb-dependent chromatin modification and is associated with poor prognosis in colorectal cancers. Cancer Res. (2011) 71:6320–6. doi: 10.1158/0008-5472.CAN-11-1021, PMID: 21862635

[8] Qiu M Xu Y Wang J Zhang E Sun M Zheng Y. A novel lncrna, luadt1, promotes lung adenocarcinoma proliferation via the epigenetic suppression of P27. Cell Death Dis. (2015) 6:e1858. doi: 10.1038/cddis.2015.203, PMID: 26291312 PMC4558496

[9] Kim K Jutooru I Chadalapaka G Johnson G Frank J Burghardt R. Hotair is a negative prognostic factor and exhibits pro-oncogenic activity in pancreatic cancer. Oncogene. (2013) 32:1616–25. doi: 10.1038/onc.2012.193, PMID: 22614017 PMC3484248

[10] Lorenzi L Avila Cobos F Decock A Everaert C Helsmoortel H Lefever S. Long noncoding rna expression profiling in cancer: challenges and opportunities. Genes Chromosomes Cancer. (2019) 58:191–9. doi: 10.1002/gcc.22709, PMID: 30461116

[11] Salmena L Poliseno L Tay Y Kats L Pandolfi PP. A cerna hypothesis: the rosetta stone of a hidden rna language? Cell. (2011) 146:353–8. doi: 10.1016/j.cell.2011.07.014, PMID: 21802130 PMC3235919

[12] Panzitt K Tschernatsch MMO Guelly C Moustafa T Stradner M Strohmaier HM. Characterization of hulc, a novel gene with striking up-regulation in hepatocellular carcinoma, as noncoding rna. Gastroenterology. (2007) 132:330–42. doi: 10.1053/j.gastro.2006.08.026, PMID: 17241883

[13] Jin C Shi W Wang F Shen X Qi J Cong H. Long non-coding rna hulc as a novel serum biomarker for diagnosis and prognosis prediction of gastric cancer. Oncotarget. (2016) 7:51763–72. doi: 10.18632/oncotarget.10107, PMID: 27322075 PMC5239513

[14] Ou Z-L Luo Z Lu Y-B. Long non-coding rna hulc as a diagnostic and prognostic marker of pancreatic cancer. World J Gastroenterol. (2019) 25:6728–42. doi: 10.3748/wjg.v25.i46.6728, PMID: 31857775 PMC6920662

[15] Shaker OG Senousy MA Elbaz EM. Association of rs6983267 at 8q24, hulc rs7763881 polymorphisms and serum lncrnas ccat2 and hulc with colorectal cancer in Egyptian patients. Sci Rep. (2017) 7:16246. doi: 10.1038/s41598-017-16500-4, PMID: 29176650 PMC5701156

[16] Zhou L Li H Sun T Wen X Niu C Li M. Hulc targets the igf1r-pi3k-akt axis in trans to promote breast cancer metastasis and cisplatin resistance. Cancer Lett. (2022) 548:215861. doi: 10.1016/j.canlet.2022.215861, PMID: 35981570

[17] Xu Y Li J Wang P Zhang Z Wang X. Lncrna hulc promotes lung squamous cell carcinoma by regulating ptpro via nf-κb. J Cell Biochem. (2019) 120:19415–21. doi: 10.1002/jcb.29119, PMID: 31448453

[18] Li Y Liu J-J Zhou J-H Chen R Cen C-Q. Lncrna hulc induces the progression of osteosarcoma by regulating the mir-372-3p/hmgb1 signalling axis. Mol Med. (2020) 26:26. doi: 10.1186/s10020-020-00155-5, PMID: 32188407 PMC7081592

[19] Chen S Wu D-D Sang X-B Wang L-L Zong Z-H Sun K-X. The lncrna hulc functions as an oncogene by targeting atg7 and itgb1 in epithelial ovarian carcinoma. Cell Death Dis. (2017) 8:e3118. doi: 10.1038/cddis.2017.486, PMID: 29022892 PMC5682654

[20] Li D Wang R Wu N Yu Y. Lncrna hulc as a potential predictor of prognosis and clinicopathological features in patients with digestive system tumors: A meta-analysis. Aging (Albany NY). (2022) 14:1797–811. doi: 10.18632/aging.203903, PMID: 35183058 PMC8908940

[21] Gao X Yang J Wang D Zeng Q Li F Zhou S. Association between hulc rs7763881 and cancer risk: an updated meta-analysis. Nucleosides Nucleotides Nucleic Acids. (2022) 41:85–96. doi: 10.1080/15257770.2021.2008433, PMID: 34865614

[22] Hämmerle M Gutschner T Uckelmann H Ozgur S Fiskin E Gross M. Posttranscriptional destabilization of the liver-specific long noncoding rna hulc by the igf2 mrna-binding protein 1 (Igf2bp1). Hepatology. (2013) 58:1703–12. doi: 10.1002/hep.26537, PMID: 23728852

[23] Wang YF Zhang S Li XQ Wang Y. Expression of lncrna hulc in cervical cancer and its correlation with tumor progression and patient survival. Eur Rev Med Pharmacol Sci. (2016) 20:3987–91., PMID: 27775802

[24] Xin X Wu M Meng Q Wang C Lu Y Yang Y. Long noncoding rna hulc accelerates liver cancer by inhibiting pten via autophagy cooperation to mir15a. Mol Cancer. (2018) 17:94. doi: 10.1186/s12943-018-0843-8, PMID: 29895332 PMC5998602

[25] Liu T Liu Y Wei C Yang Z Chang W Zhang X. Lncrna hulc promotes the progression of gastric cancer by regulating mir-9-5p/myh9 axis. BioMed Pharmacother. (2020) 121:109607. doi: 10.1016/j.biopha.2019.109607, PMID: 31726371

[26] Ghafouri-Fard S Esmaeili M Taheri M Samsami M. Highly upregulated in liver cancer (Hulc): an update on its role in carcinogenesis. J Cell Physiol. (2020) 235:9071–9. doi: 10.1002/jcp.29765, PMID: 32372477

[27] Sharma A Chowdhury S Mukherjee S Chowdhury R. Lncrna hulc augments high glucose-associated pancreatic cancer progression and drug resistance by enhancing yap activity and autophagy. Biol Cell. (2024) 116:e2400034. doi: 10.1111/boc.202400034, PMID: 38949568

[28] Li D Y-m S Zhan Q-m. Specifically up-regulated non-coding rna gene hulc in tumor cell lines and its effects on the expression of neighboring gene slc35b3. Zhonghua Yi Xue Za Zhi. (2010) 90:3156–9., PMID: 21211351

[29] Wang J Liu X Wu H Ni P Gu Z Qiao Y. Creb up-Regulates Long Non-Coding Rna, Hulc Expression through Interaction with Microrna-372 in Liver Cancer. Nucleic Acids Res. (2010) 38:5366–83. doi: 10.1093/nar/gkq285, PMID: 20423907 PMC2938198

[30] Wang C Jiang X Li X Song S Meng Q Wang L. Long noncoding rna hulc accelerates the growth of human liver cancer stem cells by upregulating cyclind1 through mir675-pkm2 pathway via autophagy. Stem Cell Res Ther. (2020) 11:8. doi: 10.1186/s13287-019-1528-y, PMID: 31900225 PMC6942366

[31] Cui M Xiao Z Wang Y Zheng M Song T Cai X. Long noncoding rna hulc modulates abnormal lipid metabolism in hepatoma cells through an mir-9-mediated rxra signaling pathway. Cancer Res. (2015) 75:846–57. doi: 10.1158/0008-5472.CAN-14-1192, PMID: 25592151

[32] Wang C Li Y Yan S Wang H Shao X Xiao M. Interactome analysis reveals that lncrna hulc promotes aerobic glycolysis through ldha and pkm2. Nat Commun. (2020) 11:3162. doi: 10.1038/s41467-020-16966-3, PMID: 32572027 PMC7308313

[33] Jiang X Wang L Xie S Chen Y Song S Lu Y. Long noncoding rna meg3 blocks telomerase activity in human liver cancer stem cells epigenetically. Stem Cell Res Ther. (2020) 11:518. doi: 10.1186/s13287-020-02036-4, PMID: 33256840 PMC7706068

[34] Xie H Ma H Zhou D. Plasma hulc as a promising novel biomarker for the detection of hepatocellular carcinoma. BioMed Res Int. (2013) 2013:136106. doi: 10.1155/2013/136106, PMID: 23762823 PMC3674644

[35] Gaber DA Shaker O Younis AT El-Kassas M. Lncrna hulc and mir-122 expression pattern in hcc-related hcv Egyptian patients. Genes (Basel). (2022) 13(9):1669. doi: 10.3390/genes13091669, PMID: 36140836 PMC9498747

[36] Yan C Wei S Han D Wu L Tan L Wang H. Lncrna hulc shrna disinhibits mir-377-5p to suppress the growth and invasion of hepatocellular carcinoma *in vitro* and hepatocarcinogenesis *in vivo* . Ann Transl Med. (2020) 8:1294. doi: 10.21037/atm-20-5556, PMID: 33209874 PMC7661872

[37] Lumkul L Jantaree P Jaisamak K Wongkummool W Lapisatepun W Orrapin S. Combinatorial gene expression profiling of serum hulc, hotair, and uca1 lncrnas to differentiate hepatocellular carcinoma from liver diseases: A systematic review and meta-analysis. Int J Mol Sci. (2024) 25(2):1258. doi: 10.3390/ijms25021258, PMID: 38279264 PMC10816616

[38] Li J Wang X Tang J Jiang R Zhang W Ji J. Hulc and linc00152 act as novel biomarkers in predicting diagnosis of hepatocellular carcinoma. Cell Physiol Biochem. (2015) 37:687–96. doi: 10.1159/000430387, PMID: 26356260

[39] Sonohara F Inokawa Y Hayashi M Yamada S Sugimoto H Fujii T. Prognostic value of long non-coding rna hulc and malat1 following the curative resection of hepatocellular carcinoma. Sci Rep. (2017) 7:16142. doi: 10.1038/s41598-017-16260-1, PMID: 29170515 PMC5700934

[40] Yao Y Duan C Chen Y Hou Z Cheng W Li D. Long non-coding rna detection based on multi-probe-induced rolling circle amplification for hepatocellular carcinoma early diagnosis. Anal Chem. (2023) 95:1549–55. doi: 10.1021/acs.analchem.2c04594, PMID: 36598887

[41] Du Y Kong G You X Zhang S Zhang T Gao Y. Elevation of highly up-regulated in liver cancer (Hulc) by hepatitis B virus X protein promotes hepatoma cell proliferation via down-regulating P18. J Biol Chem. (2012) 287:26302–11. doi: 10.1074/jbc.M112.342113, PMID: 22685290 PMC3406714

[42] Jiang Z Liu H. Metformin inhibits tumorigenesis in hbv-induced hepatocellular carcinoma by suppressing hulc overexpression caused by hbx. J Cell Biochem. (2018) 119:4482–95. doi: 10.1002/jcb.26555, PMID: 29231260

[43] Liu Y Feng J Sun M Yang G Yuan H Wang Y. Long non-coding rna hulc activates hbv by modulating hbx/stat3/mir-539/apobec3b signaling in hbv-related hepatocellular carcinoma. Cancer Lett. (2019) 454:158–70. doi: 10.1016/j.canlet.2019.04.008, PMID: 30981758

[44] Wang Y Chen F Zhao M Yang Z Li J Zhang S. The long noncoding rna hulc promotes liver cancer by increasing the expression of the hmga2 oncogene via sequestration of the microrna-186. J Biol Chem. (2017) 292:15395–407. doi: 10.1074/jbc.M117.783738, PMID: 28765279 PMC5602398

[45] Zhang H Liao Z Liu F Su C Zhu H Li Y. Long noncoding rna hulc promotes hepatocellular carcinoma progression. Aging (Albany NY). (2019) 11:9111–27. doi: 10.18632/aging.102378, PMID: 31645479 PMC6834430

[46] Liu S Huttad L He G He W Liu C Cai D. Long noncoding rna hulc regulates the nf-κb pathway and represents a promising prognostic biomarker in liver cancer. Cancer Med. (2023) 12:5124–36. doi: 10.1002/cam4.5263, PMID: 36213936 PMC9972175

[47] Xiong H Ni Z He J Jiang S Li X He J. Lncrna hulc triggers autophagy via stabilizing sirt1 and attenuates the chemosensitivity of hcc cells. Oncogene. (2017) 36:3528–40. doi: 10.1038/onc.2016.521, PMID: 28166203

[48] Xiong H Li B He J Zeng Y Zhang Y He F. Lncrna hulc promotes the growth of hepatocellular carcinoma cells via stabilizing cox-2 protein. Biochem Biophys Res Commun. (2017) 490:693–9. doi: 10.1016/j.bbrc.2017.06.103, PMID: 28634076

[49] Li S-P Xu H-X Yu Y He J-D Wang Z Xu Y-J. Lncrna hulc enhances epithelial-mesenchymal transition to promote tumorigenesis and metastasis of hepatocellular carcinoma via the mir-200a-3p/zeb1 signaling pathway. Oncotarget. (2016) 7:42431–46. doi: 10.18632/oncotarget.9883, PMID: 27285757 PMC5173146

[50] Li D Liu X Zhou J Hu J Zhang D Liu J. Long noncoding rna hulc modulates the phosphorylation of yb-1 through serving as a scaffold of extracellular signal-regulated kinase and yb-1 to enhance hepatocarcinogenesis. Hepatology. (2017) 65:1612–27. doi: 10.1002/hep.29010, PMID: 28027578

[51] Li Y Ge C Feng G Xiao H Dong J Zhu C. Low dose irradiation facilitates hepatocellular carcinoma genesis involving hulc. Mol Carcinog. (2018) 57:926–35. doi: 10.1002/mc.22813, PMID: 29573465

[52] Wang X Mo X Yang Z Zhao C. Qntrolling the lncrna hulc-tregs-pd-1 axis inhibits immune escape in the tumor microenvironment. Heliyon. (2024) 10:e28386. doi: 10.1016/j.heliyon.2024.e28386, PMID: 38560250 PMC10979100

[53] Zhang Y Song X Wang X Hu J Jiang L. Silencing of lncrna hulc enhances chemotherapy induced apoptosis in human gastric cancer. J Med Biochem. (2016) 35:137–43. doi: 10.1515/jomb-2015-0016, PMID: 28356873 PMC5346790

[54] Esfandi F Salehnezhad T Taheri M Afsharpad M Hafez AA Oskooei VK. Expression assessment of a panel of long non-coding rnas in gastric Malignancy. Exp Mol Pathol. (2020) 113:104383. doi: 10.1016/j.yexmp.2020.104383, PMID: 31982396

[55] Xian H-P Zhuo Z-L Sun Y-J Liang B Zhao X-T. Circulating long non-coding rnas hulc and znfx1-as1 are potential biomarkers in patients with gastric cancer. Oncol Lett. (2018) 16:4689–98. doi: 10.3892/ol.2018.9199, PMID: 30197680 PMC6126347

[56] Wang B-G Ding H-X Lv Z Xu Q Yuan Y. Interaction of hulc polymorphisms with helicobacter pylori infection plays a strong role for the prediction of gastric cancer risk. Future Oncol. (2020) 16:1997–2006. doi: 10.2217/fon-2020-0228, PMID: 32941073

[57] Zhao Y Guo Q Chen J Hu J Wang S Sun Y. Role of long non-coding rna hulc in cell proliferation, apoptosis and tumor metastasis of gastric cancer: A clinical and *in vitro* investigation. Oncol Rep. (2014) 31:358–64. doi: 10.3892/or.2013.2850, PMID: 24247585

[58] Yang D Shi M You Q Zhang Y Hu Z Xu J. Tumor- and metastasis-promoting roles of mir-488 inhibition via hulc enhancement and ezh2-mediated P53 repression in gastric cancer. Cell Biol Toxicol. (2023) 39:1341–58. doi: 10.1007/s10565-022-09760-y, PMID: 36449143

[59] Takahashi K Ota Y Kogure T Suzuki Y Iwamoto H Yamakita K. Circulating extracellular vesicle-encapsulated hulc is a potential biomarker for human pancreatic cancer. Cancer Sci. (2020) 111(1):98–111. doi: 10.1111/cas.14232, PMID: 31715081 PMC6942436

[60] Takahashi K Koyama K Ota Y Iwamoto H Yamakita K Fujii S. The interaction between long non-coding rna hulc and microrna-622 via transfer by extracellular vesicles regulates cell invasion and migration in human pancreatic cancer. Front Oncol. (2020) 10:1013. doi: 10.3389/fonc.2020.01013, PMID: 32656089 PMC7324724

[61] Pang Q Huang S Wang H Cao J. Hulc-igf2bp2 interaction drives proliferation and metastasis in colorectal cancer. J Cancer. (2024) 15:6686–97. doi: 10.7150/jca.101989, PMID: 39668820 PMC11632991

[62] Yang X-J Huang C-Q Peng C-W Hou J-X Liu J-Y. Long noncoding rna hulc promotes colorectal carcinoma progression through epigenetically repressing nkd2 expression. Gene. (2016) 592:172–8. doi: 10.1016/j.gene.2016.08.002, PMID: 27496341

[63] Xie X Wang Y Zhang S Li J Yu Z Ding X. A novel five-lncrna signature panel improves high-risk survival prediction in patients with cholangiocarcinoma. Aging (Albany NY). (2021) 13:2959–81. doi: 10.18632/aging.202446, PMID: 33472169 PMC7880389

[64] Wang W-T Ye H Wei P-P Han B-W He B Chen Z-H. Lncrnas H19 and hulc, activated by oxidative stress, promote cell migration and invasion in cholangiocarcinoma through a cerna manner. J Hematol Oncol. (2016) 9:117. doi: 10.1186/s13045-016-0348-0, PMID: 27809873 PMC5093965

[65] Parkin DM Bray F Ferlay J Pisani P. Global cancer statistics, 2002. CA Cancer J Clin. (2005) 55(2):74–108. doi: 10.3322/canjclin.55.2.74, PMID: 15761078

[66] Su W Tang J Wang Y Sun S Shen Y Yang H. Long non-coding rna highly up-regulated in liver cancer promotes epithelial-to-mesenchymal transition process in oral squamous cell carcinoma. J Cell Mol Med. (2019) 23:2645–55. doi: 10.1111/jcmm.14160, PMID: 30677230 PMC6433680

[67] Jiang X Liu W. Long noncoding rna highly upregulated in liver cancer activates P53-P21 pathway and promotes nasopharyngeal carcinoma cell growth. DNA Cell Biol. (2017) 36:596–602. doi: 10.1089/dna.2017.3686, PMID: 28445086

[68] Baili E Gazouli M Lazaris AC Kanavidis P Boura M Michalinos A. Genetic impact of hotair, linc00951, polr2e and hulc polymorphisms in histopathological and laboratory prognostic factors in esophageal cancer in the west: A case-control study. Cancers (Basel). (2024) 16(3):537. doi: 10.3390/cancers16030537, PMID: 38339289 PMC10854877

[69] Baili E Gazouli M Lazaris AC Kanavidis P Boura M Michalinos A. Associations of long non-coding rnas hotair, linc00951, polr2e and hulc polymorphisms with the risk of esophageal and esophagogastric junction cancer in a western population: A case-control study. Mol Biol Rep. (2024) 51:249. doi: 10.1007/s11033-024-09206-0, PMID: 38300349 PMC10834655

